# A high-resolution crossover landscape in *Drosophila santomea* reveals rapid and concerted evolution of multiple properties of crossing over control

**DOI:** 10.1371/journal.pgen.1011885

**Published:** 2025-10-06

**Authors:** Ana Llopart, Nikale Pettie, Abigail Ryon, Josep M. Comeron

**Affiliations:** 1 Department of Biology, The University of Iowa, Iowa City, Iowa, United States of America; 2 Interdisciplinary Graduate Program in Genetics, The University of Iowa, Iowa City, Iowa, United States of America; University of Georgia, UNITED STATES OF AMERICA

## Abstract

Crossing over is a fundamental process in sexually reproducing species, ensuring proper chromosome segregation during gamete formation and generating new allelic combinations that enhance adaptation. Despite its essential role, genes involved in crossing over evolve rapidly and there is extensive variation in the rate and genomic distribution of crossovers across species. Considering this rapid evolution, identifying differences between very closely related species is crucial for understanding the molecular basis of natural variation in crossing over control. Here, we present a genome-wide, high-resolution crossover map for *Drosophila santomea* and compare it with those of its sister species *D. yakuba* and the more distantly related *D. melanogaster*. Upon examining 784 individual meiotic products based on an experimental design that captures intraspecific variation in crossing over control, we identified 2,288 crossovers genome-wide. Our analyses reveal striking differences in crossover patterns between *D. santomea* and *D. yakuba* despite their recent split only 400,000 years ago and sharing a significant amount of ancestral polymorphism. The *D. santomea* X chromosome shows a major reduction in genetic length compared to *D. yakuba* (62.7 cM *vs*. 93.8 cM), while autosomes show a slight increase (262.6 *vs.* 245.6 cM), resulting in overall genetic maps of 324.2 cM for *D. santomea* and 339.3 cM for *D. yakuba*. All *D. santomea* autosomal arms show a significant reduction of the centromere effect relative to *D. yakuba,* more closely resembling *D. melanogaster* autosomes. At the same time, estimates of crossover interference indicate weaker intensity across all autosomal arms in *D. santomea* compared to *D. yakuba*, while the X chromosome exhibits considerably stronger interference. These findings suggest a link between the intensity of crossover interference and the centromere effect. We propose that stronger crossover interference is associated with a smaller crossover-competent region—determined by the combined centromere and telomere effects—to prevent the deleterious consequences of multiple crossovers occurring too close together. Finally, we examined whether the *D. santomea* X chromosome exhibits the crossover-associated meiotic drive mechanism (MD_CO_) reported in *D. yakuba*, in which chromatids with crossovers are preferentially included in oocytes. Tetrad analysis of the *D. santomea* X chromosome revealed no evidence of an active MD_CO_, potentially explaining the reduced crossover rates observed on this chromosome relative to *D. yakuba* even though the numbers of meiosis I crossovers may be similar in both species.

## Author summary

Crossovers are points of DNA exchange between maternal and paternal chromosomes that are evolutionarily vital to the generation of genetic diversity and critical for proper chromosome segregation during meiosis. Despite their biological importance, the rate and distribution of crossovers across genomes vary between species and the causes of such variation are not yet fully understood. The study of closely related species showing significant differences in crossing over control offers a promising approach to gain functional and evolutionary insights. Here, we report genome-wide, high-resolution, experimental crossover maps in *Drosophila santomea*, and compare them with those in its sister species *D. yakuba* and the more distant *D. melanogaster*. Our study shows multiple and concerted differences in crossing over control properties between *D. santomea* and *D. yakuba* despite their recent divergence only ~0.4 million years ago, suggesting extremely fast evolution. We put forward the possibility that two of these properties, centromere effect and crossover interference, are either coevolving or responding to overlapping molecular pathways. *D. santomea* also shows a significantly reduced crossover rate on the X chromosome compared to *D. yakuba*. This difference is potentially linked to recombination modifiers at the level of crossover-associated meiotic drive, effectively changing recombination rates. The rapid evolution of traits associated with crossing over control suggests that they are likely susceptible to frequent convergent evolution, which may warrant caution when interpreting comparisons between species and inferring ancestry and conservation based on similarities.

## Introduction

In sexually reproducing species, meiosis is the specialized reductional division that produces haploid cells after one round of DNA replication and two rounds of segregation [reviewed in [1,2]]. Meiotic recombination begins with developmentally programmed double strand breaks (DSBs), which are generated throughout the genome by the evolutionarily conserved topoisomerase-derived Spo11 (mei-W68 in *Drosophila*) and associated proteins [3–8]. The repair of meiotic DSBs typically results in either crossovers (CO), involving reciprocal exchange of genetic material between homologous chromosomes, or non-crossover gene conversion events (NCOGC), where the exchange is unidirectional, with a small fraction of DSBs being repaired by alternative pathways (reviewed in [7,9–13]).

Crossing over plays a fundamental role in meiosis, as it ensures that pairs of homologs remain physically tethered, facilitating their attachment to opposite poles of the meiotic spindle and ensuring proper segregation during anaphase I [9,14–19]. This direct and mechanistic effect explains why most species exhibit at least one crossover per pair of homologous chromosomes (a property termed crossover assurance) [10,20–22]. At the same time, an increased likelihood of genomic instability and improper meiotic segregation is observed in chromosome arms with multiple chiasmata, thus setting an upper bound to the number of crossovers per chromosome pair [23].

Crossovers are not randomly distributed along chromosomes. On a broad scale, this variation is largely driven by two main phenomena, the centromere effect and crossover interference (reviewed in [24–26]). The centromere effect describes the strong exclusion of crossovers near centromeres whereas crossover interference occurs when the presence of one crossover alters the probability of another crossover happening nearby. A third phenomenon, known as the telomere effect, describes the exclusion of crossovers near telomeres, although it is often less pronounced than the centromere effect and more variable among species.

The centromere effect, first identified in *D. melanogaster*, has also been detected in many other species, including *Saccharomyces cerevisiae*, *Neurospora crassa*, *Arabidopsis thaliana*, and humans [27–36]. Other than in *Drosophila*, the telomere effect has also been reported in yeast, *Caenorhabditis elegans*, and plant and mammalian female meiosis [25,33,35,37,38]. Failure to inhibit crossovers near centromeres, and to a lesser degree telomeres, increases the risk of chromosomal missegregation during meiosis, resulting in aneuploid gametes [10,19,25,39–41]. However, the ultimate causes driving the centromere and telomere effects are not yet fully understood [40–43]. Worth noting, nearly all studies formally define the centromere effect based on an observed reduction in crossovers in centromere-proximal euchromatin, where crossovers can still be identified, thus adding some ambiguity to the actual distance from centromeres given that the amount of repetitive pericentromeric heterochromatin can vary extensively among chromosomes and species [17,24,33–36,44,45].

Despite being evolutionarily conserved traits, the magnitude of the centromere and telomere effects is highly variable, even among *Drosophila* species [46–50]. For example, within the *D. melanogaster* species subgroup, a classic study using estimates of crossover rates based on P-element markers showed reduced centromere and telomere effects in *D. mauritiana* relative to *D. melanogaster*, with the two species sharing a common ancestor 5.4 million years ago (Mya) [46]. This finding has been recently confirmed by Hawley *et al*. (2025) using genome-wide, SNP-based crossover maps [51]. In *D. yakuba*, a species that diverged from *D. melanogaster* 12.8 Mya, the centromere effect is remarkably stronger than that in *D. melanogaster* [50]. *D. virilis*, a distant outgroup to the *D. melanogaster* subgroup that last shared a common ancestor with *D. melanogaster* and *D. yakuba* more than 60 Mya [52], also shows reduced centromere and telomere effects compared to *D. melanogaster* [53]. These results suggest no clear phylogenetic signal and indicate that the genetic control of the centromere effect may evolve rapidly in *Drosophila*.

Another mechanism that regulates the location of crossovers across genomes is crossover interference. In the case of positive crossover interference, multiple crossovers along the same chromatid are spaced farther apart than expected if they formed independently. Positive crossover interference was first identified in *Drosophila* [54,55] and is now considered to be present in most, though not all, eukaryotes [56–60]. In the *Drosophila* genus, positive crossover interference has been well-characterized in *D. melanogaster* [61], *D. yakuba* [50], *D. virilis* [53], and in a 3Mb region on the **D. pseudoobscura* XR* chromosome arm [62]. However, there is ample variation in the intensity of interference across species, with humans, dogs, *D. melanogaster* and yeast all exhibiting differences. An extreme case of positive interference is *C. elegans*, where each chromosome pair typically has exactly one crossover [60]. On a smaller evolutionary scale, *D. yakuba* shows stronger interference than *D. melanogaster* [50,61]. Ultimately, positive crossover interference plays a role controlling crossover location along chromosomes and, in extreme cases, limits crossover number as well. Notably, high-resolution recombination analyses in *D. melanogaster* have shown that NCOGC events are impervious to interference, detectable in regions with reduced or absent crossovers, and not affected by centromere and telomere effects [61,63].

Together, the centromere effect and crossover interference are key components for ensuring proper chromosome segregation while limiting the number of crossovers per chromosome to a remarkably small range of 1–3 in most species, despite large differences in chromosome size. Because chromosome and genome size can change rapidly between species, the relative conservation in the number of crossovers per chromosome arm suggests that the mechanisms controlling crossover number and location are either coevolving and/or responding to the same or overlapping pathways. Supporting the latter possibility, *D*. *melanogaster Blm* (Bloom syndrome helicase) mutants lack both the centromere effect and crossover interference [64].

Within the large chromosomal regions modulated by the centromere and telomere effects as well as crossover interference, there is also ample variation in crossover rates at a smaller scale, often in the form of hotspots [65–70]. In many species with a strong signature of hotspots, such as birds, dogs, plants and yeasts, crossovers are enriched in promoter regions and tend to be evolutionarily stable [71–81]. On the other hand, in most mammals—including humans and mice, but not canids—hotspots are driven by the zinc finger protein PRDM9, which binds to fast-evolving DNA motifs preferentially located in intergenic regions [82–86]. *Drosophila* lack PRDM9, and there is no evidence of *bona fide* recombination hotspots. However, analyses of high-resolution crossover maps have shown that crossovers are overrepresented at actively transcribed genes and near simple, non-satellite DNA motifs [47,48,50,53,63,87]. Specifically, DNA motifs that show a strong effect on crossover rate variation across the *D. melanogaster* genome share properties associated with open chromatin and high DNA accessibility [88]. Given the different factors driving large- and fine-scale variation, the degree of conservation in the chromosomal distribution of crossovers depends on the scale of analysis, with more conserved properties when analyzing crossover distribution at large scales (>1Mb) [50,63,89–92].

The exchange of genetic material between parental genomes via meiotic recombination is also an important evolutionary process for sexually reproducing species [93–99]. The new allelic combinations introduced during meiotic recombination can uncouple the evolutionary trajectories of segregating alleles, whether neutral or under selection. This dynamic interplay between natural variation, selection, and recombination drives adaptation and is one of the pervasive advantages of sexual reproduction and meiotic recombination over asexual reproduction. The same population-level dynamics apply across genomes, leading to a positive association between intragenomic crossover rate variation and both levels of neutral nucleotide diversity and efficacy of selection [93,100–106]. While recombination and crossing over are sometimes used interchangeably, it is primarily crossovers that drive the functional and evolutionary benefits of meiotic recombination, with NCOGC events playing a secondary role.

Despite the critical role of crossovers in evolution and chromosome segregation, crossover rates are also variable within species in a wide range of taxa, including *Drosophila*, humans, sheep, mice, snails, maize and *Arabidopsis* [63,107–116]. This high level of natural variation suggests that crossing over control (both rates and patterning) could itself be subject to selection [63,83,90,107,110,117–123]. Supporting this idea, multiple studies in *Drosophila* have shown that recombination rates can increase very rapidly in response to selection, both directly to change recombination rates or indirectly through experiments targeting fitness-related traits [107,121,122,124–131]. Moreover, analyses of intraspecific natural variation suggest that selection may influence seasonal and geographic variation in crossover rates [108,110]. The high degree of crossover rate variation within species also indicates that crossing over control is likely a polygenic trait [122,126,132]. In line with this possibility, studies of natural variation in crossover rates in *D. melanogaster* and *D. pseudoobscura* have identified multiple loci associated with heritable variation in recombination rates [109,110,130,133].

The molecular basis of variation in crossover rate and patterning between species is less understood, and the study of closely related species with notable differences offers a promising opportunity. Brand *et al*. (2018) demonstrated that interspecific differences in the dicistronic gene *mei-217/mei-218* are causally responsible for variation in crossover rate and distribution between *D. melanogaster* and *D. mauritiana* [134]. In a later study, the same group showed that sequence divergence between *mei-218* alleles from *D. pseudoobscura*, which diverged from *D. melanogaster* more than 50 Mya, and *D. virilis* also had functional consequences for crossover patterning in a *D. melanogaster* genomic background [135]. Despite these advances, studies of interspecific variation between species with much closer evolutionary relationships would further help to characterize the causes of crossing over control evolution.

Pettie *et al*. (2022) recently generated genome-wide, high-resolution crossover maps in *D. yakuba* using a novel and efficient dual-barcoding genotyping approach, which also captures intraspecific variation [50]. This study revealed contrasting patterns of crossover rates and distribution compared to *D. melanogaster,* with *D. yakuba* showing higher crossover rates, a stronger centromere effect, and more intense crossover interference. In fact, the centromere effect in *D. yakuba* is the strongest identified in the *Drosophila* genus to date. Interestingly, *D. yakuba* departs from other *Drosophila* species in which an increase in crossovers is usually coupled with a reduction in centromere effect. Instead, *D. yakuba* shows both a higher crossover rate and a stronger centromere effect. Furthermore, tetrad analysis for the X chromosome uncovered crossover-associated meiotic drive (MD_CO_) in *D. yakuba* that preferentially includes chromatids with crossovers in the oocyte pronucleus relative to their non-recombinant sister chromatids. MD_CO_ is different from the standard female meiotic drive associated with centromeres that takes place during meiosis I [136–138] in that it occurs during meiosis II instead. As such, MD_CO_ could constitute an example of the non-centromeric drive of the oötid competition model [139]. Notably, this form of meiotic drive would effectively provide the evolutionary benefits of high recombination rates for offspring while minimizing chromosome missegregation due to multiple crossovers during meiosis [50,140].

In this study, we generated the first high-resolution, genome-wide meiotic crossover map of *D. santomea* and compared it to its sister species *D. yakuba* and the more distantly related *D. melanogaster*. *D. yakuba* and *D. santomea* split from their most recent common ancestor only ~400,000 years ago [141–143]. Despite this short divergence time, the two species show several reproductive barriers along with morphological and behavioral differences [142,144–154]. Our analyses reveal multiple differences between *D. santomea* and *D. yakuba* in both crossover rates and distribution across chromosome arms, evidencing very rapid evolution of recombination patterns and control. *D. santomea* shows a markedly reduced centromere effect compared to *D. yakuba,* more closely resembling *D. melanogaster*. For autosomes, this reduction in centromere effect is associated with a reduction in the intensity of crossover interference and longer genetic maps relative to *D. yakuba*. These results support a model in which the intensity of crossover interference increases when the crossover-competent region of a chromosome decreases. At the same time, the X chromosome of *D. santomea* shows no signal of the MD_CO_ observed in *D. yakuba* and a 33% reduction in overall crossover rates. Because *D. santomea* and *D. yakuba* produce mostly fertile F_1_ hybrid females and they hybridize in the laboratory as well as in nature [141,155–159], the large differences in crossover rates and patterning between these two species provide a unique opportunity for future studies to dissect the molecular genetic basis of crossing over control evolution.

## Results

To generate a high-resolution, whole-genome crossover map for *D. santomea*, we utilized the dual-barcoding methodology described in Pettie *et al*. (2022) (see Materials and Methods for details). Our experimental design allows capturing intraspecific variation in crossover number and distribution, and we created crossover maps for eight different crosses using 16 parental wild-type isofemale lines (see **Table 1** and Materials and Methods). Heterozygous F1 females from each of these eight crosses were mated with males from an additional parental line (tester) to produce F_2_ individuals, which were sequenced and genotyped to identify crossover locations. To this end, we first obtained high-quality sequences for the 17 *D. santomea* parental lines used in the crossing scheme (Materials and Methods for details). These sequences enabled us to identify SNPs specific to each line (singletons), which can be used as diagnostic SNPs to remove genotypic information from the tester line and localize crossovers following Pettie *et al*. (2022). On average, each parental genome provided more than 51,000 diagnostic SNPs genome-wide, with an average of one diagnostic SNP per 2 kb.

**Table 1:** *D. santomea* parental lines and crosses used in this study.

	Lines^1^
Cross 1	*Thena 8* × *Obat 1200.14*
Cross 2	*Rain 41* × *CAR 1600.2*
Cross 3	*Quija 18* × *Rain 37*
Cross 4	*Quija 14* × *Field 1*
Cross 5	*Thena 5* × *Rain 40*
Cross 6	*Rain 34* × *Quija 1*
Cross 7	*Thena 2* × *STO.4*
Cross 8	*Field 6* × *Thena 7*
Tester	CAR 1566.5

^1^ For each cross, the line corresponding to the female parent is listed first*.*

In total, we genotyped 784 F_2_ individuals using Illumina whole-genome sequencing (WGS), each representing a distinct meiotic event (see Materials and Methods for details). A polymorphic chromosomal inversion was identified on chromosome arm 2R and five out eight crosses produced F_1_ individuals heterozygous for the inversion (Materials and Methods). Consequently, data from 2R were excluded from the analyses unless otherwise noted. Heterozygous inversions influence crossing over within the inverted region and can also increase rates elsewhere in the genome, including other chromosome arms (i.e., the interchromosomal effect; [160,161]). We investigated whether there was a detectable interchromosomal effect due to the presence of the heterozygous 2R inversion. The number of crossovers outside chromosome arm 2R was not significantly higher in crosses with the heterozygous inversion compared to those without it (*F*-statistic = 2.10, *P* = 0.20). The absence of a detectable interchromosomal effect in *D. santomea*, at least for the inversion identified on 2R, aligns with similar findings in *D. yakuba* [50] and *D. simulans* [162].

### Genetic map and crossover distribution in *D. santomea*

**Fig 1:**
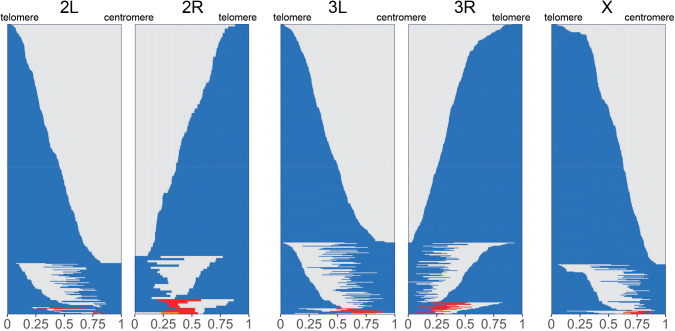
Relative location of crossovers along chromosome arms in *D. santomea.* Each horizontal line represents a genotyped chromosome with one or more crossovers. A change in color indicates the location of a crossover. Within each chromosome arm, chromosomes have been ordered based on crossover class for better visualization, from 1 crossover (top) to 3 or 4 crossovers (bottom). Within each crossover class, genotyped chromosomes are ordered by their most telomere-proximal crossover position, closer to the telomere at the top, progressing towards the centromere, at the bottom.

**Fig 2:**
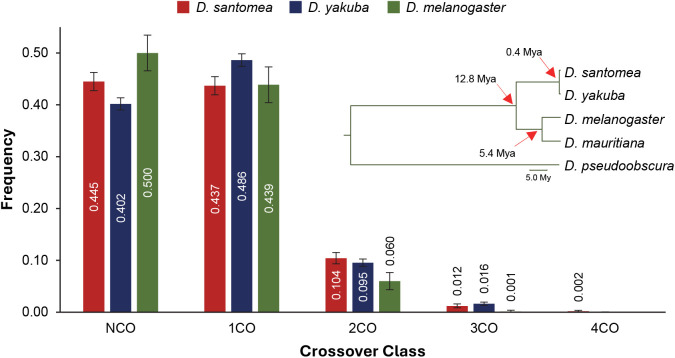
Observed frequency of different meiotic events in *D. santomea*, *D. yakuba*, and *D. melanogaster*. *D. melanogaster* data is from Miller *et al.* (2016) and *D. yakuba* data is from Pettie *et al.* (2022). NCO, zero crossovers; 1CO, single CO; 2CO, double CO; 3CO, triple CO; 4CO, quadruple CO in a single chromatid. Error-bars indicate 95% confidence intervals. The inset depicts a simplified phylogeny of the species mentioned in the main text with divergence times. Data shown after excluding chromosome arm 2R in all species for comparative purposes.

Overall, we identified 2,288 crossover events in *D. santomea.* **Fig 1** shows the relative locations of these crossovers along the five major chromosome arms. We recovered a total of 1,523 arms with zero crossovers (NCOs), 1,451 with a single crossover (1COs), 340 with two crossovers (2COs), 43 with three crossovers (3COs), and 7 with four crossovers (4COs) (**Table 2**). Comparative analysis of these crossover classes in *D. santomea*, *D. yakuba* and *D. melanogaster* shows that the frequency of arms with no crossovers in *D. santomea* (44.5%) falls between that in *D. yakuba* (40.2%) and *D. melanogaster* (50.0%). The frequency of chromatids with multiple crossovers (2CO, 3CO and 4CO) in *D. santomea* and *D. yakuba* is similarly high (11.8% *vs*. 11.2%, respectively), and nearly doubles the figure observed in *D. melanogaster* (6.1%) (**Fig 2**). We did not detect any crossovers on chromosome 4, which is consistent with the lack of chiasmata reported on this dot chromosome in other *Drosophila* species [64,163–166].

**Table 2:** Observed number of meiotic events in *D. santomea.*

Crossover Class^1^	Chromosome				
	2L	2R^2^	3L	3R	X
NCO	375	154	346	294	354
1CO	336	107	329	348	331
2CO	62	20	99	95	64
3CO	10	6	7	15	5
4CO	0	1	2	4	0
Total	783	288	783	756	754

^1^ NCO: Zero crossovers, 1CO: single crossover, 2CO: double crossover, 3CO: triple crossover, 4CO: quadruple crossover in a single chromatid.

^2^ There were fewer individuals analyzed in chromosome arm 2R because several crosses generated F_1_ individuals heterozygous for a 2R inversion.

Our study estimates an average of 3.34 crossovers per viable meiotic product in *D. santomea*, which is comparable to the 3.46 in *D. yakuba* [50] and 21% higher than the 2.76 estimated in *D. melanogaster* [63]. Likewise, the genome-wide crossover rates (cM/Mb) and total genetic map length (cM) in *D. santomea* (2.61 cM/Mb and total 324.2 cM) are comparable to those in *D. yakuba* (2.76 cM/Mb and total 339.3 cM) but significantly higher than in *D. melanogaster* (2.10 cM/Mb and 277.3 cM) (**Table 3**). However, a more detailed comparative analysis reveals a strikingly different pattern for autosomes and the X chromosome. While crossover rates for autosomal arms are similar in the two sister species—albeit slightly higher in *D. santomea* than in *D. yakuba* (2.57 *vs*. 2.41 cM/Mb, respectively)—the crossover rate on the *D. santomea* *X* chromosome shows a substantial decrease compared to that of *D. yakuba* (2.71 *vs*. 4.07 cM/Mb, respectively)*.* As a result, the *X* chromosome accounts for only 19.3% of the entire *D. santomea* recombination map and generates an X-to-autosome (X/A) ratio of crossover rates of 1.05. This finding contrasts with the disproportionate contribution of the *X* chromosome to genome-wide crossover rates in both *D. yakuba* (27.6% of the total genetic map and X/A ratio of 1.69) and *D. melanogaster* (24% of the total genetic map and X/A ratio of 1.36) (**Table 3**). The significant difference in crossover rate on the X chromosome between *D. santomea* and *D. yakuba* is particularly remarkable given the short evolutionary time since their divergence and the pervasive presence of ancestral shared polymorphism.

**Table 3:** Genetic maps and crossover rates estimated for *D. santomea*, *D. yakuba*, and *D. melanogaster.*

Chromosome arm	Genetic Map (cM)			Average Crossover Rate (cM/Mb)		
	*D.santomea*	*D.yakuba*	*D.melanogaster*	*D.santomea*	*D.yakuba*	*D.melanogaster*
2L	62.51	57.22	55.33	2.70	2.47	2.40
2R	59.80	55.23	56.44	2.69	2.51	2.66
3L	61.55	71.28	44.08	2.39	2.76	1.79
3R	78.72	61.82	54.96	2.54	2.00	1.96
Autosomal arms	262.58	245.55	210.81	2.57	2.41	2.17
X	62.66	93.77	66.45	2.71	4.07	2.95
All	324.24	339.32	277.28	2.61	2.76	2.10

Data for *D. yakuba* from Pettie *et al*. (2022) and data for *D. melanogaster* (r5) from Comeron *et al*. (2012).

At a local scale, we investigated the presence of short DNA motifs identified as enriched near crossovers in both *D. melanogaster* and *D. yakuba* [50,88]. Our analysis shows that the same short DNA motifs are also significantly enriched near crossovers in *D. santomea* (S1 Table; see Materials and Methods for details). These motif classes include [A]_N_, [CA]_N_, [TA]_N_, which are associated with open chromatin through secondary and tertiary DNA structures [88,167,168]. This conservation indicates that some aspects of fine-scale crossover localization in *Drosophila* species qualitatively align with crossover hotspot data from *S. cerevisiae* [71,169,170].

**Fig 3:**
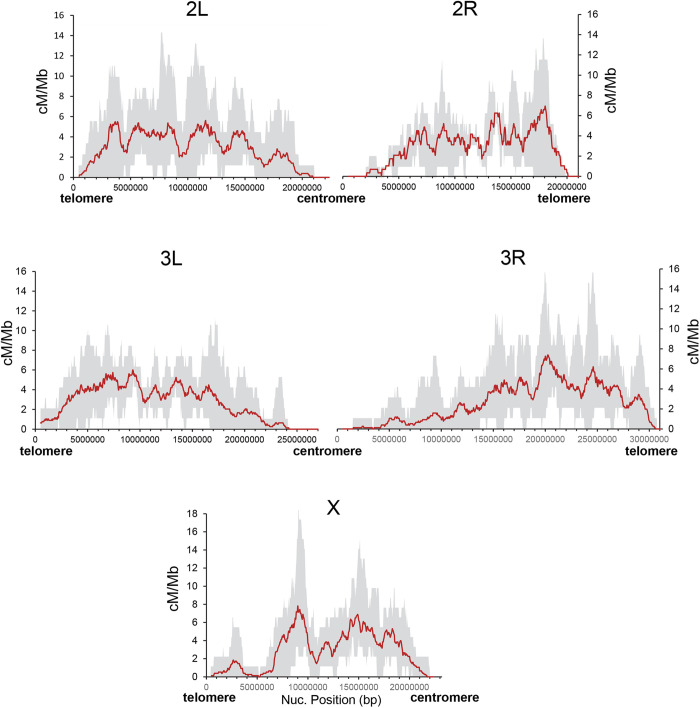
Crossover rate distribution in *D. santomea* for all major chromosome arms. Average crossover rate in cM/Mb per female meiosis (red line) shown for 1-Mb overlapping windows with increments of 50 kb. Grey area depicts the highest and lowest crossover rates among the eight crosses analyzed.

Genome-wide, our experimental design and the large number of genotyped individuals allowed us to generate a fine-resolution *D. santomea* crossover landscape that also captures intraspecific variation (**Fig 3**). We identify highly variable crossover rates along chromosomes, with regions showing estimates up to 17cM/Mb for specific crosses when analyzed at 1-Mb scale. The average rate from the eight different crosses also shows significant heterogeneity along chromosomes, up to 7.43 cM/Mb when analyzed at 1-Mb scale (12.7 cM/Mb when analyzed at 250-kb scale) (**Figs 3** and S1). Direct comparison between crossover landscapes in *D. santomea* and *D. yakuba* identifies significant similarity at multiple scales, with chromosome arm 3R showing the strongest rank association across scales (S2 Fig). This analysis also indicates that the conservation of crossover landscapes along chromosomes decreases at fine scales, particularly below 500kb, in agreement with the proposal of different modes of variation in crossover regulation at fine and broad scales, the latter being likely more conserved due to selection on mechanisms of chromosome segregation [91,92].

### Autosomes in *D. santomea* show strongly reduced centromere effect relative to *D. yakuba*

**Fig 4:**
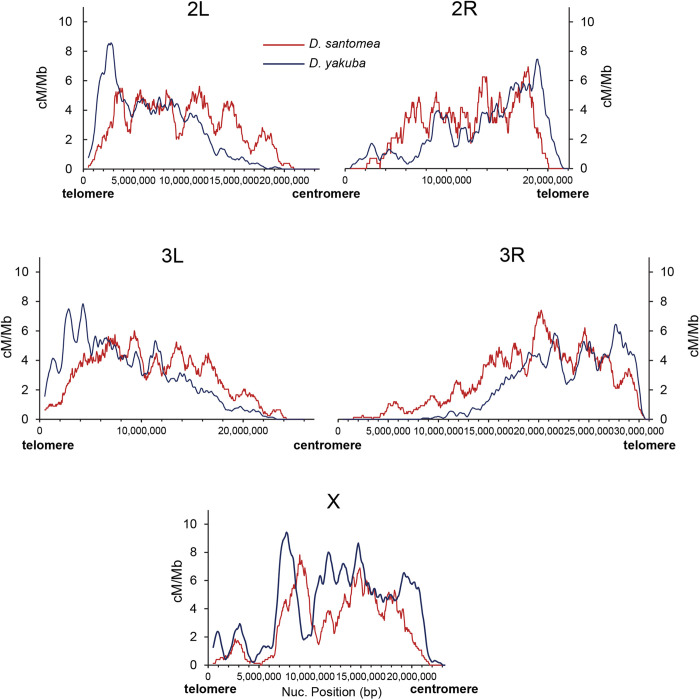
Comparison of high-resolution crossover maps between *D. santomea* and *D. yakuba.* For each species, the average crossover rate (cM/Mb) from different crosses is shown along the major chromosome arms (1-Mb overlapping windows with 50 kb increments). Data for *D. yakuba* was obtained from Pettie *et al*. (2022).

A visual comparison of the crossover landscapes indicates that *D. santomea* has weaker centromere effect on autosomes than *D. yakuba* (Fig 4). To quantitatively evaluate this difference, we applied two approaches. First, we used the standard method of analyzing a centromere-proximal region of arbitrary size, defined here as one-third of the chromosome arm (as in [61]), and compared the observed number of crossovers in that region to the number expected if crossovers were randomly distributed along the chromosome arm [17,33,36,61]. This analysis indicates a significant centromere effect in all chromosome arms analyzed in *D. santomea* (*P* < 1 × 10^-6^ for X, 2L, 3L and 3R) while *D. yakuba* showed significance only for autosomes [50]. When applying the same approach to telomere-proximal regions, no significant crossover reduction was detected in any of the *D. santomea* telomeres (**P* *> 0.50 in all cases), whereas *D. yakuba* showed a significant reduction only for the X chromosome [50].

**Fig 5:**
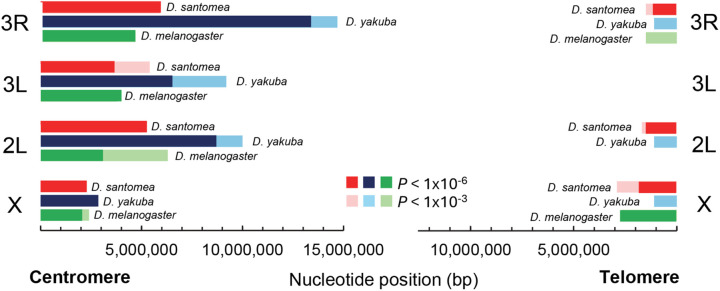
The centromere/telomere effect in *D. santomea*, *D. yakuba* and *D. melanogaster.* Size (in bp) of the genomic region experiencing the centromere- or telomere-effect. Estimates of the centromere and telomere effects for *D. yakuba* and *D. melanogaster* based on subsampling to generate equivalent numbers of crossovers per chromosome arm in all three species (see text and S2 Table for details). Darker colors indicate significance at *P* < 1x10^-6^ and lighter colors indicate significance at **P* *< 1x10^-3^.

To avoid the dependency on the arbitrary size of the centromere- and telomere-proximal regions, we also applied an alternative method described in Pettie *et al*. (2022) that directly estimates the size of the region of a chromosome arm showing a statistically significant reduction in crossovers. This approach allows for quantitative comparisons of the centromere or telomere effect across chromosomes or species. We note, however, that the number of meioses analyzed in *D. yakuba* [50] and *D. melanogaster* [63] was higher than in *D. santomea* and that differences in the total number of crossovers would generate a difference in statistical power. To assess centromere effects with a comparable number of crossovers for each chromosome arm, we performed subsampling of *D. yakuba* and *D. melanogaster* crossover data (Fig 5 and S2 Table for results before and after subsampling). This analysis revealed that *D. santomea* has a significantly weaker centromere effect than its sister species *D. yakuba*, almost exclusively due to differences on autosomes. At *P* < 10^-6^ significance, the size of the region significantly impacted by centromere effects in *D. santomea* is reduced by a total of 12.5 Mb relative to *D. yakuba*, with the proportion of the genome affected decreasing from 27% in *D. yakuba* to 15% in *D. santomea.* At the same time, *D. santomea* autosomes show similar centromere effects as those in *D. melanogaster*. All three species show a similarly weak centromere effect on chromosome X. The telomere effect is weaker than the centromere effect in all *D. santomea* autosomes (Fig 5 and S2 Table), as it is the case in *D. yakuba* and *D. melanogaster* [17,46,50,61,63]. Interestingly, the use of comparable crossover numbers to assess telomere effects suggests that *D. santomea* has stronger telomere effects than *D. yakuba* and *D. melanogaster*. The magnitude of this difference, however, is modest (less than 3Mb).

**Fig 6:**
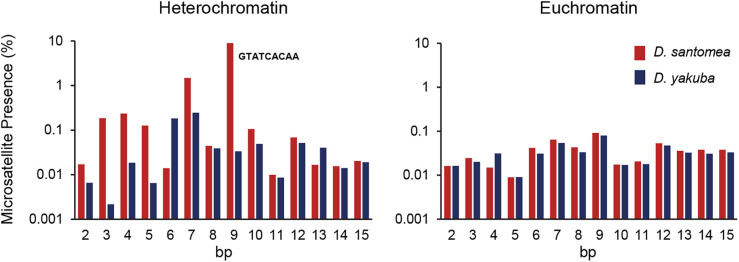
Microsatellite arrays in heterochromatic and euchromatic sequences of *D. santomea* and *D. yakuba.* Percentage (log_10_ scale) of sequence covered by tandem arrays of microsatellite repeats ranging between 2 and 15-bp.

An explanation for the observed difference in centromere effect between *D. santomea* and *D. yakuba* is larger pericentromeric heterochromatin regions in *D. santomea*, effectively increasing the distance between the functional centromere and the most centromere-proximal euchromatic regions analyzed in genetic maps [35,36,50,51,171–173]. To investigate this possibility, we applied a two-pronged approach (see Materials and Methods). First, we used the fraction of Illumina reads from parental genomes that do not map to our mostly euchromatic *D. santomea* (this study) and *D. yakuba* [50] reference genomes as an indirect measure of the fraction of heterochromatic, mostly pericentromeric, regions in these species. After examining multiple strains for each species, this analysis shows a significantly larger fraction of unmapped reads in *D. santomea* (average 0.19) than in *D. yakuba* (average 0.12) (Mann-Whitney U test, *P* = 0.000007; see Materials and Methods). Second, to identify differences in repeat arrays between *D. santomea* and *D. yakuba*, we analyzed sequence properties of long PacBio reads that map and do not map to the reference genomes (see Materials and Methods). This analysis shows a similar presence of microsatellite repeat arrays along euchromatic sequences of both species, but a substantial overrepresentation in the heterochromatic sequences of *D. santomea* relative to *D. yakuba* (with a 17-fold higher density of microsatellite arrays in *D. santomea*; **Fig 6** and S3 Table), suggesting longer pericentromeric alpha-heterochromatin in *D. santomea*. Notably, *D. santomea* heterochromatic sequences are particularly enriched in arrays of a 9-nt (GTATCACAA) microsatellite relative to either *D. yakuba* or *D. melanogaster*, indicating a very recent expansion.

### Crossover interference has diverged between *D. santomea* and *D. yakuba*

To investigate crossover interference in *D. santomea*, we estimated the observed inter-crossover distance (ICD) in 2CO chromatids and compared it with the expected ICD under no interference, calculated from the distribution of crossovers in 1CO chromatids (see Materials and Methods). Importantly, this approach to estimate the expected ICD accounts for the effects that centromeres and telomeres have reducing the genomic space available to crossovers as well as the potential impact of a heterogeneous distribution of crossovers along a chromosome arm. All *D. santomea* autosomal arms and the X chromosome show positive crossover interference when analyzed individually (Table 4). The genome-wide expected ICD under no interference is 5,199 kb whereas the observed ICD is 8,594 kb, consistent with positive interference (*P* = 0.009; Table 4).

**Table 4:** Crossover interference in **D. santomea*.*

	X	2L	3L	3R	All^2^
Number of chromatids with 2COs	64	62	99	95	340
Mean inter-crossover distance (ICD; kb)	7,839	7,672	8,409	10,236	8,594
Minimum ICD (kb)	1,168	591	2,872	1,677	591
Expected ICD (kb)^1^	4,823	5,609	5,918	6,492	5,199
Ratio Observed/Expected ICD	1.625	1.368	1.421	1.577	1.653
Prob (Observed ≥ Expected ICD)	< 1 x 10^–6^	2 x 10^–6^	< 1 x 10^–6^	< 1 x 10^–6^	0.009
Expected *ν* (gamma) for ICD	2.02	2.05	1.96	1.83	1.89
Observed *ν* (gamma) for ICD in 2COs	7.22	3.61	5.60	2.05	3.40

^1^ Expected ICD based on the distance between two crossovers from randomly chosen chromatids with a single crossover (1CO) (see text for details).

^2^ Includes data from chromosome arm 2R.

We also assessed crossover interference based on the full distribution of observed ICD in 2CO chromatids and the estimated shape parameter (*ν*) when fitting the ICDs to a gamma distribution [59,174,175]. Estimates of *ν* under this model of crossover interference provide a measure of the intensity of interference. Under random distribution of crossovers along chromosomes and no interference, *v* is expected to be 1, and *ν* greater than 1 indicates positive crossover interference. However, because crossovers (including 1COs) are not randomly distributed along chromosome arms, expectations for *v* need to be calculated based on pairs of randomly chosen 1COs, which are chromosome and species-specific (see Materials and Methods).

In *D. santomea*, all autosomal arms and the X chromosome show positive crossover interference, with observed *v* greater than expected (Table 4). Genome-wide, our estimate of *v* indicates a reduction in the intensity of crossover interference in *D. santomea* (*v* = 3.40) relative to *D. yakuba* (*v* = 4.90). The overall weaker intensity of crossover interference in *D. santomea* is due to autosomes, with *v* of 3.97 and 6.35 for *D. santomea* and *D. yakuba*, respectively. For the X chromosome, on the other hand, the intensity of crossover interference is much higher in *D. santomea* (*v* = 7.22) than in *D. yakuba* (*v* of 3.87). Interestingly, changes in the intensity of crossover interference between *D. santomea* and *D. yakuba* are accompanied by changes in expected ICD. Autosomes showed decreased intensity of crossover interference in *D. santomea* relative to *D. yakuba* while the expected ICD is greater in the former species (5,834 kb) than in the latter (5,360 kb). The X chromosome, which experiences more intense crossover interference in *D. santomea* than in *D. yakuba*, shows smaller expected ICD in *D. santomea* (4,823 kb) than in *D. yakuba* (6,059 kb). Within species, the *D. santomea* X chromosome shows the highest *v* and the smallest expected ICD of all chromosome arms (Table 4), whereas in *D. yakuba* the X exhibits the lowest *v* and the largest expected ICD. Taken together, our analyses of crossover interference in *D. santomea* and *D. yakuba* reveal multiple consistent signals of an inverse relationship between expected ICD and the intensity of crossover interference, both across chromosome arms and in interspecific comparisons of these sister species.

### Tetrad analysis and crossover assurance

**Fig 7:**
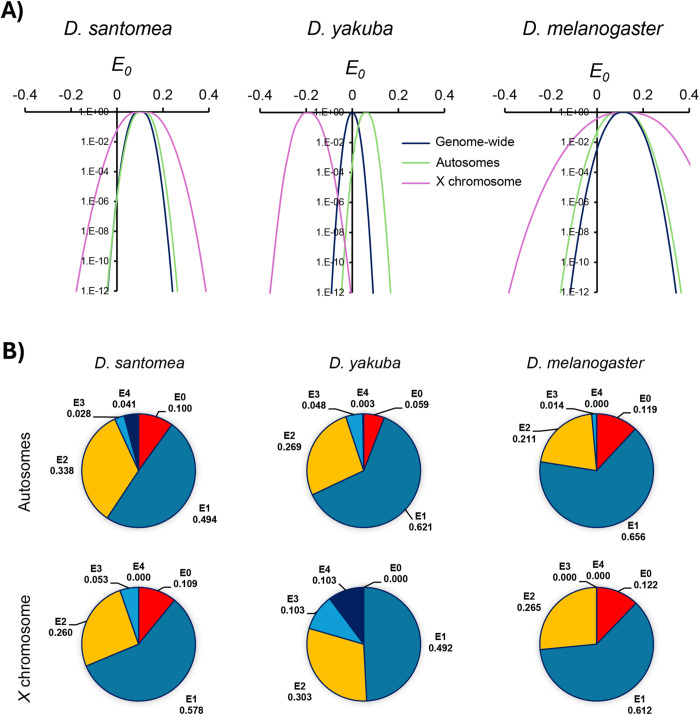
Estimates of tetrad frequencies. **A)** Probability of tetrad models to fit the observed crossover classes across a range of *E*_0_, with *E*_*>0*_ restricted to biologically feasible values (1 ≥* *E**_*>0 *_≥ 0). For each *E*_*0*_, the best combination of *E*_*>0*_ was estimated by the random search method. Probabilities shown relative to the best point estimate for *E*_0_. **B)** Autosomal (top half) and X chromosome (bottom half) estimates of tetrad frequencies based on models that constrain frequencies within a biologically feasible range (*E*_*r*_ ≥ 0). For *D. santomea*, *D. melanogaster* and the autosomes of *D. yakuba*, the best model is equivalent to an unrestricted model and shows a good fit with the data. The best model for the X chromosome of *D. yakuba* is *E*_*0 *_= 0, but nonetheless incompatible with the observed data for this species (*P*[*E*_*0 *_= 0] < 1 x 10^-12^). Data to estimate tetrad frequency for *D. yakuba* and *D. melanogaster* from Pettie *et al*. (2022) and Miller *et al.* (2016), respectively. *E*_0_: tetrads that do not undergo crossing over, *E*_1_: tetrads with 1 CO, *E*_2_: tetrads with 2 COs, *E*_3_: tetrads with 3 COs, and *E*_4_: tetrads with 4 COs.

We used Weinstein’s model to estimate the frequency of different tetrad classes in *D. santomea* and compared them to *D. yakuba* and *D. melanogaster*. This method allows estimating *E*_*r*_ (where *r* indicates the number of crossovers per tetrad) for models with a variety set of rules (see and Materials and Methods for details). We first applied a direct model with unrestricted range for *E*_*r*_ under ideal conditions of random distribution of crossovers among chromatids, equal viability of meiotic products, random chromatid distribution into gametes, and the absence of both crossover and chromatid interference (Fig 7A). Genome-wide, both *D. santomea* and *D. melanogaster* show estimates of *E*_*0*_ significantly greater than 0, while *D. yakuba* shows estimates very close to 0, suggesting a stronger degree of overall crossover assurance. Estimates of *E*_0_ for autosomes are similar across all three species. However, the estimate of *E*_*0*_ for the *D. yakuba* X chromosome forecasts a negative value (see [50]), while *E*_0_ in *D. santomea* (0.109) is equivalent to that in *D. melanogaster* (0.122). When applying a model with biologically feasible restrictions to *E*_r_ (1 ≥ *E*_*r*_ ≥ 0), the best model for the X chromosome of *D. yakuba* shows *E*_*0*_ = 0, and the overall frequency of tetrad classes in *D. santomea* more closely resembles estimates for *D. melanogaster* than for *D. yakuba* (Fig 7B). These results are unexpected given the close evolutionary relationship between *D. santomea* and *D. yakuba,* emphasizing the notion that control of crossover number can evolve very rapidly, at least on the X chromosome, and provide little information in terms of evolutionary relationships.

## Discussion

Comparative studies of crossover rate and patterning provide an opportunity to identify the genetic and genomic factors responsible for natural variation in the control of crossing over, which may be influenced by natural selection. Although crossing over is known to be a highly polygenic trait, as evidenced by studies of intraspecific variation and mutant surveys, few genes have been identified as responsible for differences in broad-scale patterning—such as the centromere effect and crossover interference—between species. Within the *Drosophila* genus, *mei-218*, has been found to directly contribute to interspecific variation in crossover patterning, with evidence of recurrent positive selection for amino acid changes [134,135]. With notable exceptions (see [110,176]), a key limitation in previous comparative studies has been the relatively high divergence between the species analyzed, making it a challenge to identify the causes of crossover patterning changes. Additionally, only experimentally-based crossover maps would allow the simultaneous study of the multiple properties of crossing over control, including crossover interference. Therefore, the examination of crossover properties in very closely related species holds particular interest for understanding crossing over control.

We recently generated a high-resolution crossover map for *D. yakuba* [50], and in this study we describe the map for its sister species *D. santomea*, which diverged from *D. yakuba* only 400,000 years ago [141,142]. Importantly, our crossing scheme and whole-genome analysis of individual meiotic events in *D. santomea* allowed us to capture potential intraspecific variation in crossing over patterning. This map, therefore, provides the unique opportunity to study variation in crossing over control between species with low nucleotide sequence divergence that still share ancestral polymorphism and hybridize in the laboratory as well as in nature. We also compared the high-resolution crossover map of *D. santomea* and *D. yakuba* to that of *D. melanogaster*, which serves as outgroup to the *D. santomea*-*D. yakuba* comparison within the *D. melanogaster* subgroup.

Genome-wide, *D. santomea* shows a slightly shorter genetic map than *D. yakuba* (324.2 vs 339.3 cM, respectively). Relative to other *Drosophila* species with empirical genetic maps, *D. santomea* has a map of intermediate length, longer than *D. melanogaster* (277 cM [61,63,177]), and shorter than *D. mauritiana* (about 500 cM [46]), *D. pseudoobscura* (>450 cM [178,179]), *D. virilis* (732 cM [53]) and *D. ananassae* (962 cM [180]). Notably, the variation in genetic maps between *D. santomea* and *D. yakuba* shows a stark difference between autosomes and the X chromosome. Relative to *D. yakuba*, *D. santomea* autosomes show a slight increase in genetic map (262.6 vs 245.6 cM in *D. santomea* and *D. yakuba*, respectively) whereas the X chromosome shows a major reduction (62.7 vs 93.8cM in *D. santomea* and *D. yakuba*, respectively).

The centromere effect is a wide-spread phenomenon in *Drosophila*, with *D. yakuba* showing the strongest effect (up to 47% of the chromosome arm 3R with reduced crossover frequency) while *D. mauritiana* is possibly an exception [17,33,34,36,46,50,51,63,181]. Consistent with other *Drosophila* species, *D. santomea* also shows a reduction in crossover rates near centromeres across all chromosome arms and the X chromosome (Figs 4 and 5). Our analyses reveal that *D. santomea* displays a significantly smaller genomic region of crossover exclusion compared to *D. yakuba*. Specifically, more than 18 Mb influenced by the centromere effect in *D. yakuba* show no crossover suppression in *D. santomea*. Interestingly, the centromere effect in *D. santomea* more closely resembles that of *D. melanogaster* than that of *D. yakuba*, despite the more distant evolutionary relationship between *D. santomea* and *D. melanogaster.* Like in other *Drosophila* species [17,50,61,63,172], the X chromosome of *D. santomea* exhibits the weakest centromere effect across chromosome arms, and it is of equivalent magnitude to that in the X chromosome of *D. yakuba* and *D. melanogaster* (Fig 5).

Although the mechanisms mediating the centromere effect in *Drosophila* are not fully understood, several areas of research provide key insights. There is evidence suggesting that crossovers are suppressed by both highly repetitive heterochromatin and proximity to the centromere (in *cis*) with little, if any, *trans* effects [173,182]. These observations are consistent with early work indicating that the ability to detect centromere effects can be enhanced by deleting large amounts of proximal heterochromatin, which decreases the distance between the functional centromere and the most centromere-proximal genomic regions used for mapping [172]. In this regard, our analyses suggest, albeit indirectly, a difference in the amount of pericentromeric heterochromatin between *D. santomea* and *D. yakuba*, with more extensive highly repetitive alpha-heterochromatin in *D. santomea*, thus potentially explaining the reduced centromere effect relative to *D. yakuba*.

Given the high rate of evolution of heterochromatic sequences and the difference in satellite composition between *D. santomea* and *D. melanogaster*, the apparent similarity in centromere effect between these two species (both with longer pericentromeric regions and reduced centromere effect than *D. yakuba*) could be the result of convergent evolution. The differences in centromere effect between *D. santomea*, *D. yakuba* and *D. melanogaster*, therefore, could be explained, at least in part, by neutral processes driving differences in the amount of pericentromeric alpha-heterochromatin. However, recent studies have also shown a contribution of *trans* factors altering the centromere effect in *D. melanogaster*, including synaptonemal complex proteins such as c(3)G [183,184]. This opens the possibility of coevolution of structural properties of pericentromeric sequences and regulatory pericentromeric or synaptonemal proteins, which could be driven by positive selection in a manner that parallels the evolution of functional centromeres and *Cid* or the 359-bp satellite DNA and *mh* in the *melanogaster* subgroup [136–138,185,186]. Additional genetic and genomic studies designed to characterize the full structure of the centromeres, peri-centromeric regions and the interacting proteins in multiple closely related species are needed to assess the evolutionary forces driving changes in centromere effect across species.

In the context of meiotic crossover patterning, the centromere effect has also been linked to other phenomena, including crossover interference [24,59]. Centromeres, and potentially other spatial domains including telomeres, have been proposed to act as “sinks” of stress relief, pushing crossovers away and toward the medial regions of the chromosome arms, thus influencing patterning and potentially increasing crossover interference [64,187,188]. In *D. melanogaster* Hatkevich *et al*. (2017) reported that a loss of function mutation in the Bloom Syndrome Helicase gene (*Blm*) severely weakens both centromere effects and crossover interference, suggesting interdependency [64]. Similarly, Brand *et al*. showed that the wild-type allele of the gene *mei-217/mei-218* from multiple *Drosophila* species simultaneously reduces the magnitude of the centromere effect and the intensity of crossover interference in *D. melanogaster* transgenic flies [134,135]. However, Brady *et al.* (2018) demonstrated that interference can be reduced in the absence of changes in centromere effect in *mei-41* mutants, which led the authors to propose a stepwise model in which the two processes are independent and temporally separated [189]. Considering the striking difference in centromere effect between *D. santomea* and *D. yakuba* (Fig 5 and S2 Table) and the close evolutionary relationship between these two species, our data allow investigating the connection between the different types of patterning events under conditions of similar meiotic chromosomal structures.

The overall intensity of interference, estimated from the shape parameter *ν*, is lower in *D. santomea* than in *D. yakuba*, in parallel with weaker centromere effects in *D. santomea.* More relevant to the potential mechanistic causes of crossover interference, the weaker intensity of interference in *D. santomea* is associated with greater genome-wide expected ICD, which we use as a proxy for the crossover-competent region on chromosomes. The autosomes in *D. santomea* show greater expected ICD and weaker intensity of interference than autosomes in *D. yakuba*. Conversely, the X chromosome of *D. santomea* shows smaller expected ICD and stronger intensity of crossover interference than in *D. yakuba*. When comparing the chromosome arms of *D. santomea*, the chromosomes with the smallest (4,823 kb for the X chromosome) and largest expected ICD (6,492 kb for the chromosome arm 3R), correspond to the strongest (*v* of 7.22 for the X chromosome) and weakest (*v* of 2.05 for the chromosome arm 3R) intensity of crossover interference, respectively.

Together, our data support a mechanistic two-step model in which the intensity of crossover interference increases when the crossover-competent region of a chromosome decreases. The size of this crossover-competent region would be determined by a combination of centromere and telomere effects, crossover distribution and the total physical length of chromosomes, which can vary between chromosome arms and species. Strong centromere effects that cause expected ICD to be small might be necessary but not sufficient to induce crossover interference. Conversely, an increase in expected ICD due to a reduction in the centromere effect would reduce the need to keep double crossovers farther apart than expected by random chance, and the intensity of interference. Under this scenario, variants diminishing the centromere effect would simultaneously weaken both phenomena, resulting in both weaker centromere effect and crossover interference (e.g., *Blm*, *mei*-*218* or *c(3)G*). The patterns of crossover in *D. mauritiana* and *D. pseudoobscura* would align with such possibility, with weak suppression of crossovers in centromere-proximal euchromatin (and presumably greater expected ICD) and limited crossover interference [47,134,190]. On the other hand, null variants of genes required for the process of crossover interference would not need to affect the centromere effect (e.g., *mei-41* mutants). Additionally, there are few, if any, examples of species with a recent reduction in the intensity of interference that have maintained a strong centromere effect, as expected due to the strongly deleterious consequences of crossovers occurring too close to each other during meiosis.

Our results also indicate that the primary difference in crossover rates between *D. santomea* and *D. yakuba* lies on the X chromosome (62.7 cM in *D. santomea* and 93.8 cM in *D. yakuba*). Tetrad analysis of *D. santomea* reveals that the fraction of chromatids without crossovers (*E*_0_) estimated from the observed distribution of meiotic products for the X chromosome is comparable to that in *D. melanogaster* (0.11 and 0.12 for *D. santomea* and *D. melanogaster*, respectively). However, tetrad analyses for the *D. yakuba* X chromosome suggest the presence of a crossover-associated meiotic drive mechanism (MD_CO_). The proposed MD_CO_ in *D. yakuba* results in the preferential inclusion of chromatids with crossovers into the oocytes at the expense of non-recombinant sister chromatids during meiosis II. This effectively increases crossover rates in offspring without increasing actual crossover events during meiosis [50]. In essence, this mechanism amplifies the evolutionary benefits of higher recombination rates while avoiding the deleterious effects associated with higher rates of ectopic exchange and mis-segregation in multi-chiasma tetrads. Notably, a similar MD_CO_ mechanism has been previously reported in *D. melanogaster* as an epigenetic response to parasite infection [140], while the MD_CO_ in *D. yakuba* appears to function under benign conditions [50]. Our analyses of *D. santomea* show no evidence of active MD_CO_ on the X chromosome. Interestingly, estimates of *E*_0_ for *D. yakuba* when allowing MD_CO_ overlap with our estimate of *E*_0_ (0.11) for the *D. santomea* X chromosome. Therefore, the large difference in crossover number on the *D. santomea* X chromosome relative to *D. yakuba* is compatible with both species having a similar number of meiotic crossover events during prophase I, with *D. santomea* lacking MD_CO_. Future studies will determine whether the difference between species represents a recent change in the *D. santomea* lineage, in *D. yakuba*, or a combination of both.

In all, our study reveals very rapid evolution of multiple traits of crossing over control between two very closely related *Drosophila* species, including crossover rates, centromere effect and crossover interference, with consistent changes in these two latter phenomena between species and across chromosome arms. The rapid evolution of crossover properties, together with fast turnover of pericentromeric sequences and the likely coevolution of regulatory and structural components of crossover homeostasis, would indicate that crossover traits are susceptible to frequent convergent evolution. Therefore, evolutionary inferences of ancestral and derived states should be made with caution, even within the same species group. In the case of our study between *D. santomea* and *D. yakuba*, the use of *D. melanogaster* as outgroup to infer ancestry and conservation based on similarities might not be fully adequate. Instead, future studies to delve deeper into the molecular causes of the observed differences in crossing over control should focus on *D.teissieri*, which represents a much closer outgroup to the *D. santomea*-*D. yakuba* system.

The results also highlight the idiosyncrasy of the *D. yakuba* X chromosome. The *Drosophila* X chromosome experiences more intense natural selection than autosomes, leading to faster-X evolution for protein sequences and gene expression, particularly in *D. yakuba*-*D. santomea* interspecific comparisons [143,191–199]. Our data, together with results from artificial selection experiments in *D. melanogaster* [125], would suggest that the X chromosome is also more prone to evolutionary changes in crossover rates and distribution due to indirect selection on modifiers of recombination.

## Materials and methods

### Fly stocks, crossing scheme, and library preparation

To capture intraspecific variation in recombination maps, our study included 17 *D. santomea* isofemale lines (i.e., parental lines) and eight different crosses (see Table 1). All ‘Quija’ lines were derived from females collected near Rio Quija, Southwest São Tomé, in an area that also harbors *D. yakuba*. The remaining lines were established from females collected in the *D. yakuba-D. santomea* hybrid zone at the Ôbo Natural Reserve [154,200]. All flies were maintained at 24°C on a standard cornmeal-yeast-agar medium and a 12h light/dark cycle.

One-day old F_1_ virgin females were crossed to males from the tester line CAR 1566.5 and their offspring (F_2_) was sequenced to allow for the bioinformatic identification of haploid sequences along the maternally transmitted chromosomes [50,63]. To prepare Illumina libraries for genome sequencing, we followed the methods described in Comeron *et al*. (2012) and Pettie *et al*. (2022). DNA was isolated from adult flies using the Qiagen DNAeasy Blood & Tissue kit (Qiagen, Germantown, MD) and fragmented using a Bioruptor UCD-200 (Diagenode, Denville, NJ). Libraries were prepared using the NEBNext DNA Library Prep Master Set for Illumina (New England Biolabs, Ipswich, MA) following manufacturer’s recommendations, with size selection and custom-designed barcodes [50]. All samples were multiplexed using 98 barcodes prior to next generation sequencing.

### Genome sequencing of *D. santomea* lines and identification of diagnostic SNPs

We sequenced all *D. santomea* parental and the tester lines on an Illumina HiSeq 4000 platform at the Iowa Institute of Human Genetics (IIHG), Genomics Division (University of Iowa), with an overall average coverage of 35 × . To obtain the genome sequences of the parental lines and maximize read coverage, we first built a *D. santomea* synthetic genome based on the reads from all parental libraries combined sequentially (> 627 million reads) and the *D. yakuba* reference genome sequence [50]. The synthetic genome sequence not only incorporated *D. santomea* intraspecific variation at nucleotide level but was also used as scaffold to maximize the number of mapped reads when generating the genome sequences of each individual parental line following Pettie *et al*. (2022). Briefly, for each line, we used an alignment pipeline that involves two rounds of mapping to the synthetic sequence using first Bowtie2 (default settings for sensitive aligning) [201] followed by Stampy version 1.0.32 [202]. Samtools mpileup with parameters of minimum map quality 35 and base quality 30 was used to call variants [203], and BCFtools to filter out sites with less than 3 reads supporting the call and a fraction of reads calling the same variant < 80% [204]. Vcfutlis vcf2fq and seqtk were used to convert VCF files to fasta format [203]. Our approach generated genome sequences for each parental line in which specific bases were only called for sites with high-quality information. To identify diagnostic SNPs, we focused on sites that (1) had a high-quality base call in all parental genomes, including the tester, and (2) showed a different base variant in only one of the parental genomes (i.e., singleton) [50]. Heterozygous sites, sites with low quality, or sites with ambiguity in one or more parental genomes were filtered out.

### Generation of crossover maps

To generate genome-wide, high resolution crossover maps, we genotyped 784 F_2_ individuals by sequencing 98 pools of eight F_2_ flies (one fly from each cross) on a full SP flow cell (150 bp paired-end run) of an Illumina NovaSeq 6000 system (IIHG). Following Pettie *et al*. (2022) and after filtering reads from the tester library, crossovers were identified in F_2_ individuals as a switch in the origin of a block of diagnostic SNPs along the maternally transmitted chromosomes. A block was defined as a minimum of 25 consecutive diagnostic SNPs spanning a minimum of 250 kb. For the dot (4^th^) chromosome the requirement was relaxed to four consecutive diagnostic SNPs in a block to account for the lower density of polymorphisms. Furthermore, we also required that crossovers be at least 250 kb apart in 2CO chromatids. Increasing this distance to 500 kb had no effect, which is consistent with the observation that the minimum inter-crossover distance is ~ 591 kb in our *D. santomea* dataset (Table 4). Crossover rates were estimated in cM/Mb per female meiosis. All analyses comparing the distribution of crossover rates along chromosomes in *D. santomea* were carried out using non-overlapping windows unless otherwise noted. F_2_ chromosome arms with fewer than 100 diagnostic SNPs (10 diagnostic SNPs in the case of the dot chromosome) were excluded.

In this study, we were able to bioinformatically account for all chromosomal rearrangements between *D. yakuba* and *D. santomea* genomes. However, we also identified a polymorphic chromosomal inversion on chromosome arm 2R of *D. santomea* that generated heterozygous F_1_ females in five out of the eight crosses. As a result, our analyses and genetic maps are based on eight crosses except for 2R, where all analyses were restricted to three crosses. Recombination rates (cM/Mb) per female meiosis for *D. santomea* and *D. yakuba* are available in S4 Table.

### Centromere and telomere analysis

The centromere and telomere effects were evaluated using two different methods [50]. The standard method compares the number of crossovers observed in a centromere- or telomere-proximal region with the number of crossovers expected in a region of equivalent size if crossovers were randomly distributed along a chromosome arm (or genome). We assessed centromere or telomere effects in a proximal region that was one-third of the chromosome arm following Miller *et al*. (2016), and probabilities were determined based on 10 million replicates for each chromosome arm. Importantly, only chromosome regions where crossovers can be potentially detected based on our F_2_ genotyping method were considered. Although this approach has been extensively used [17,33,36,61], it is limited by the requirement of a predetermined arbitrary size of the proximal region. To overcome this limitation, Pettie *et al*. (2022) developed a method that estimates the size of the region significantly impacted by the crossover suppression near centromeres or telomeres using a sliding window analysis, with a window size of 1Mb and step increments of 100 kb. Starting with the window most proximal to the centromere or telomere, a binomial model is used to determine whether the number of observed crossovers in that window is significantly smaller than that expected under the assumption of randomly distributed crossovers. The presence of five consecutive windows showing no statistical departure from random expectations indicates the end of the genome region impacted by the centromere or telomere effects. We applied this approach with two levels of statistical significance (*P* = 1 x 10^-6^ and *P* = 0.001).

For analyses of *D. melanogaster*, we used the release *r5* of the *D. melanogaster* reference genome because it contains less heterochromatin than the most recent release, making it more comparable to our *D. santomea* template sequence, which does not contain any heterochromatin [181]. To generate comparable estimates of the magnitude of the centromere/telomere effect between species, we applied the quantitative, sliding window, approach to the full data from *D. yakuba* and *D. melanogaster* but also to subsamples of these datasets to make them equivalent in number of crossovers per chromosome arm to those from *D. santomea*.

### Detection and analysis of heterochromatin

To estimate the amount and repeat composition of heterochromatin, mostly pericentromeric, in *D. santomea* and *D. yakuba*, we followed two different approaches. For the first approach, Illumina reads from the *D. santomea* (this study) and *D. yakuba* [50] parental lines were trimmed and filtered based on quality using FASTX-Toolkit (v0.014) (http://hannonlab.cshl.edu/fastx_toolkit/). This same program was used to normalize mapping probabilities across all reads by further trimming reads to 125 bases. Mapping to *D. santomea* and *D. yakuba* genome reference sequences was carried out by Bowtie2 (v2.3.4.2) [201] in combination with SAMtools (v1.3.1) [203]. We assumed that the fraction of reads that did not map to the references, mostly euchromatin, constitutes an estimate of the fraction of heterochromatin present in the genome for each line. For our second approach, PacBio reads for *D. santomea* (NCBI SRR12282725) and *D. yakuba* (NCBI SRR12277927) were split into fragments of 5,000 bases, quality filtered using the program Filtlong (v0.2.0) (filtlong –min_length 1,000 –keep_percent 90) [205] (https://github.com/rrwick/Filtlong), and mapped to *D. santomea* and *D. yakuba* genome reference sequences using Minimap2 (v2.26-r1175) [206]. To determine potential differences in repeat presence, mapped and unmapped PacBio reads were analyzed using TRF (Tandem Repeats Finder), a program to identify tandem repeats in DNA sequences [207].

### Crossover interference

To study crossover interference in *D. santomea,* we compared the average inter-crossover distance (ICD) in 2CO chromatids with the ICD expected under no interference. To obtain an expectation for ICD, we generated a null distribution of crossover distances by randomly subsampling 1 million sets of two 1CO chromatids. The average of this distribution represents the expected ICD, and positive crossover interference is inferred when the fraction of subsamples showing crossover distances smaller than those observed in the data is less than 0.05 [208]. This approach incorporates the suppression of crossovers by centromeres and telomeres as well as any other potential genomic property associated with a non-random distribution of crossovers along chromosomes.

We also investigated the non-random distribution of crossovers in 2CO chromatids by estimating the shape parameter (*ν*) when we fit ICD to a gamma distribution [the gamma model of crossover interference [174,175]. Under a case of no interference and random location of crossovers in 2CO chromatids, the expectation for *ν* is 1. Positive crossover interference, on the other hand, predicts a gamma distribution of ICD with *ν* greater than 1 [59,174,175]. To account for variation in crossover rates along chromosomes, we obtained the expected *ν* based on the random generation of ICD using the location of two randomly chosen crossovers from 1CO chromatids.

### Tetrad analysis

To estimate the frequency of the different tetrad (or bivalent) classes, *E*_*r*_, from the observed frequency of crossover classes [zero (NCOs), one (1COs), two (2COs), three (3COs) or four (4COs) crossovers], we used Weinstein’s algebraic method, with *r* denoting the number of crossovers (e.g., *E*_*0*_ indicates the frequency of tetrads with no crossovers, *E*_1_ tetrads with a single crossover, etc…) [209,210]. Estimates of *E*_*r*_ in *D. santomea* were compared to those obtained when using crossover classes from *D. yakuba* [50] and *D. melanogaster* [61], the two *Drosophila* datasets generated with equivalent genotyping (WGS) methods. In the case of the X chromosome of *D. yakuba*, Weinstein’s direct (unrestricted) approach generates a negative value for *E*_*0*_, which indicates that the model’s underlying conditions are not met, leading to unreliable parameter estimates for all *E*_*r*_. We, therefore, identified the best combination of biologically feasible values for *E*_*r*_ (restricting *E*_*r*_ to the [0,1] range) by using Weinstein’s equations and minimizing the difference between predicted and observed crossover classes (chi-square test) with two different approaches, random search and numerical optimization (NMinimize function) in Wolfram Mathematica (v14.2). This allows identifying the best combination of restricted *E*_*r*_ and directly generating the probability to fit the observed crossover data. These approaches also allow additional restrictions (e.g., *E*_*4*_ ≤ *E*_*3*_) and the possibility of drive during meiosis II with a bias favoring chromatids with crossovers preferentially segregating into the oocyte nucleus when the sister chromatid has no or fewer crossovers (the MD_CO_ model [50]). We also used the random search and the numerical optimization methods to obtain the best combination of *E*_*1-4*_ (restricted to the [0,1] range) and the probability to fit the observed crossover classes across a range of *E*_*o*_.

### Detection of crossover motifs

To determine whether the DNA motifs significantly enriched near crossovers in *D. melanogaster* and *D. yakuba* [63,87] are also enriched near crossovers in our *D. santomea* dataset, we analyzed 5kb sequences flanking crossovers. The same number of sequences, with the same length, were generated from random genomic locations as a negative control. We used FIMO from the MEME suite [211] to identify motifs in our two datasets, and performed a chi-square test to determine whether motifs were significantly overrepresented in the *D. santomea* crossover sequences compared to the negative control. Despite the smaller sample size relative to the *D. yakuba* study [50], all motif classes analyzed showed significant enrichment.

## Supporting information

S1 Fig Crossover rate distribution in *D. santomea* at different genomic scales. (PDF)

S2 Fig Spearman’s correlation of crossover rates between *D. santomea* and *D. yakuba* at different genomic scales. (PDF)

S1 Table DNA motif enrichment near crossovers in D. santomea. (PDF)

S2 Table The centromere and telomere effect in *D. santomea*, *D. yakuba* and *D. melanogaster.* (PDF)

S3 Table Microsatellite repeat arrays in *D. santomea* and *D. yakuba.* (PDF)

S4 Table Recombination rates (cM/Mb) for *D. santomea* and *D. yakuba.* (XLSX)
