# Supplementary material for: A high-resolution crossover landscape in Drosophila santomea reveals rapid and concerted evolution of multiple properties of crossing over control
Source: PLoS Genet. 2025 Oct 6;21(10):e1011885. doi: 10.1371/journal.pgen.1011885 (PMC12500166; doi:10.1371/journal.pgen.1011885)
Supplement: S1 Fig — (PDF) [file pgen.1011885.s001.pdf]

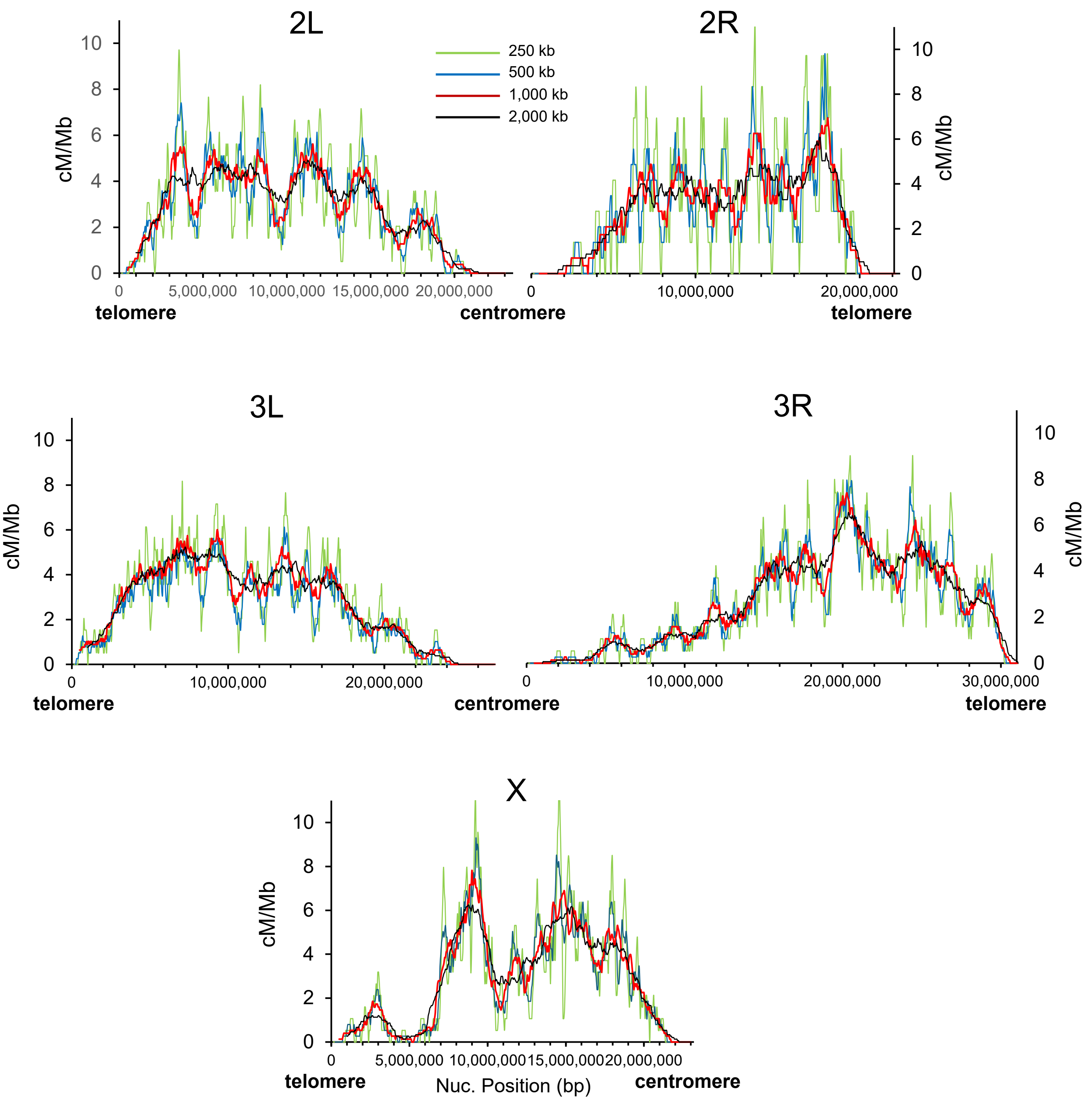

S1 Figure. Crossover rate distribution in *D. santomea* at different genomic scales.
Average crossover rate in cM/Mb per female meiosis shown for overlapping windows of variable size (from 250-kb to 2-Mb), with increments of 50 kb.
