# Supplementary material for: A high-resolution crossover landscape in Drosophila santomea reveals rapid and concerted evolution of multiple properties of crossing over control
Source: PLoS Genet. 2025 Oct 6;21(10):e1011885. doi: 10.1371/journal.pgen.1011885 (PMC12500166; doi:10.1371/journal.pgen.1011885)
Supplement: S2 Fig — (PDF) [file pgen.1011885.s002.pdf]

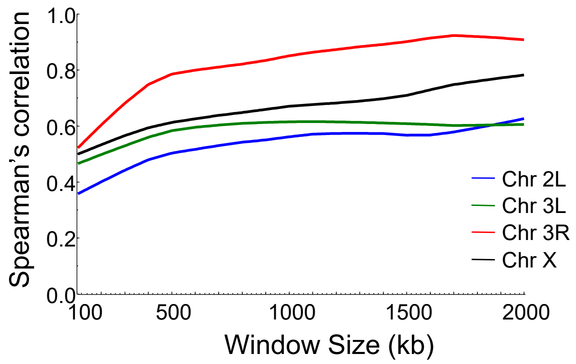


**S2 Figure.** Spearman's correlation of crossover rates between *D. santomea* and *D. yakuba* at different genomic scales. Lowess (locally weighted scatterplot smoothing) plot of Spearman's correlations for nonoverlapping windows ranging from 100 kb to 2,000 kb in increments of 100 kb.
