# Supplementary material for: A high-resolution crossover landscape in Drosophila santomea reveals rapid and concerted evolution of multiple properties of crossing over control
Source: PLoS Genet. 2025 Oct 6;21(10):e1011885. doi: 10.1371/journal.pgen.1011885 (PMC12500166; doi:10.1371/journal.pgen.1011885)
Supplement: S1 Table — (PDF) [file pgen.1011885.s003.pdf]

S1 Table. DNA motif enrichment near crossovers in *D. santomea*

Motif[1]	*P*-value[2]
$[A]_N$	3.89×10^{-14}
$[CA]_N$	8.47×10^{-51}
$[TA]_N$	0.002
$[GCA]_N$	0.008
$[CYCYYY]_N$	5.02×10^{-8}

[1] Motifs obtained from Adrian *et al*. (2016). [2] *P*-values were based on comparing the number of times a motif is present in sequences near a crossover event to expectations based on sequences of the same length (5 kb) randomly chosen from across the genome. *P*-values obtained using a χ^2 test.
