# Supplementary material for: A high-resolution crossover landscape in Drosophila santomea reveals rapid and concerted evolution of multiple properties of crossing over control
Source: PLoS Genet. 2025 Oct 6;21(10):e1011885. doi: 10.1371/journal.pgen.1011885 (PMC12500166; doi:10.1371/journal.pgen.1011885)
Supplement: S2 Table — (PDF) [file pgen.1011885.s004.pdf]

S2 Table. The centromere and telomere effect in *D. santomea*, *D. yakuba* and *D. melanogaster*

		Centromere effect (kb) [1]			
Species	Significance Level	*X*	*2L*	*3L*	*3R*
D. santomea	$P < 1{\times}10^{-6}$	2,000	4,600	3,200	5,200
	$P < 0.001$	2,000	5,000	5,400	5,900
D. yakuba	$P < 1{\times}10^{-6}$	2,700	9,900	8,900	14,500
	$P < 0.001$	2,900	11,200	9,400	15,500
D. yakuba	$P < 1{\times}10^{-6}$	2,500	7,600	5,700	11,700
(CO normalized)[2]	$P < 0.001$	2,700	10,000	9,200	14,700
D. melanogaster	$P < 1{\times}10^{-6}$	2,100	6,200	3,500	4,500
	$P < 0.001$	2,600	6,500	4,000	4,800
D. melanogaster	$P < 1{\times}10^{-6}$	1,800	2,700	3,500	4,100
(CO normalized)[2]	$P < 0.001$	2,400	6,300	3,800	4,700

		Telomere effect (kb) [1]			
Species	Significance Level	*X*	*2L*	*3L*	*3R*
D. santomea	$P < 1{\times}10^{-6}$	1,600	1,300	<1,000	1,000
	$P < 0.001$	2,900	1,700	<1,000	1,500
D. yakuba	$P < 1{\times}10^{-6}$	2,900	1,000	<1,000	1,100
	$P < 0.001$	3,300	1,200	<1,000	1,200
D. yakuba	$P < 1{\times}10^{-6}$	<1,000	<1,000	<1,000	<1,000
(CO normalized)[2]	$P < 0.001$	1,100	1,100	<1,000	1,100
D. melanogaster	$P < 1{\times}10^{-6}$	2,600	<1,000	<1,000	1,300
	$P < 0.001$	2,900	1,000	1,000	1,700
D. melanogaster	$P < 1{\times}10^{-6}$	2,400	<1,000	<1,000	<1,000
(CO normalized)[2]	$P < 0.001$	2,700	<1,000	<1,000	1,500

[1] Region showing a significant reduction in crossover events. [2] Results after subsampling of *D. yakuba* and *D. melanogaster* crossover data to generate the same number of crossovers than in *D. santomea* for each chromosome arm.
