# Supplementary material for: A high-resolution crossover landscape in Drosophila santomea reveals rapid and concerted evolution of multiple properties of crossing over control
Source: PLoS Genet. 2025 Oct 6;21(10):e1011885. doi: 10.1371/journal.pgen.1011885 (PMC12500166; doi:10.1371/journal.pgen.1011885)
Supplement: S3 Table — (PDF) [file pgen.1011885.s005.pdf]

S3 Table. Microsatellite repeat arrays in *D. santomea* and *D. yakuba*

	Presence of microsatellite repeat arrays (%)	
	Euchromatin	Heterochromatin
D. yakuba	0.460	0.722
D. santomea	0.514	11.283
